# Cast Gold Inlays

**Published:** 1908-02-15

**Authors:** Edwin E. Harcourt

**Affiliations:** Cincinnati


### CAST GOLD INLAYS.

BY EDWIN E. HARCOURT, D. D. S., CINCINNATI.

*Burs.*—In doing gold inlay work a few special instruments are required: such as square end and pointed smooth-cut fissure burs of the medium sizes, for both the straight and right angle hand-piece. Judgment should be displayed in the selection of these. Also a diamond bur, cylindrical in shape, sizes 12 and 13. If you have to buy these new, get the S. S. W. special inlay burs, such as long, cylindrical fissure burs with smooth, round ends, which keeps them from cutting too deep into cavity, or marring the walls. Good inlay burs can be made from the old-style, spear-pointed or cone-shaped bur by grinding the tips off flat. It will be necessary to get some of these for the right angle hand-piece. One plug finishing bur is needed, No. 249. This is used for serrating the back so as to make the cement adhere and also for removing little beads caused by air bubbles due to faulty investment.

*Stones.*—As for carborundum stones, it will be necessary to have a few small knife-edged stones of the smallest make; also a long, abrasive point No. 13 S. S. W., to be inserted into a port polisher. One small cylindrical Arkansas stone for polishing the edges. In a great many cases, especially where the dentine is very sensitive, the cavities may be ground to shape and finished by these small carborundum stones kept wet. By steadying the hand-piece and drawing the small round or cylindrical-shaped stones in and out of the cavity, the walls may be made perfectly true, and to flare or taper from the bottom outward. By this method the stones are made to wear perfectly true, and it is not necessary to discard them after a few usings

*Cavity Preparation.*—In many cases you will not find it necessary to separate the teeth, even in approximo-occlusal cavities. Of course, wedging is a great assistance to the operator, but under the present method of rubber, it is torture to the patient. Can't somebody suggest something superior to the method now in vogue? As in the old method you must have sufficient room in which to burnish the matrix to conform with the cavity. You will find in some cases you can bevel the approximal margins with a knife-edged stone sufficiently to allow the wax impression to be withdrawn. But should the gingival margin be covered by the gum, it will be necessary to pack it with gutta percha with sufficient pressure to cause the gum to recede slightly. The principles of cavity preparation are essential, and should be understood. In small cavities where it is desirable, sound tooth structure may be sacrificed in order to properly prepare the cavity so the wax impression will readily withdraw. But in large cavities where the teeth are badly decayed, when nothing but a shell remains, and where the cavity makes deep undercuts, in order to taper the walls, it is necessary to cut too much of the tooth away. With an execevator, clean out the cavity and wipe with a pledget of cotton saturated with carbolic acid; then wipe dry and fill the bottom and up the sides to the edge with cement. When hard, this can be shaped up with a stone or bur so the wax will draw, but be sure and shape it so that no cement remains at the edge, so the gold will lap over to the extreme edges of the faced enamel. If any sharp points or angles are left standing it would be better to round them slightly, as this not only greatly facilitates drawing of the wax impression, but also prevents little beads on the back of the inlay, as the air has a tendency to pocket in these sharp angles when investing. I am referring principally to cavities in bicuspids and molars on the occlusal or the approximo-occlusal surfaces. You will find in these teeth that the gold inlay is more practicable, especially on the occlusal surfaces which present a series of inclined planes which would be hard to restore with sufficient strength by any other method than that of the gold inlay. Should the labial or lingual walls be very thin, so that by occlusion with the opposite teeth they are likely to become fractured, shorten the thin walls and bevel the edges from the outside of the tooth to the inside or to the cavity walls, so when the inlay is made it will lap over the edge and bind so as to protect it, as you would the cutting edge of a facing in bridgework. After you have your cavity shaped, polish and slightly round and bevel the edges with a small Arkansas stone kept wet. This causes a thin feather-edge on the wax impression, which is duplicated in the gold inlay, and can be burnished down tight to the margins when set.

*Retention.*—Inlays must be made so they are self-locking, or so they mortise or wedge their way down tight when properly seated. Not so they can be pried out at any angle. In cavities of the approximo-occlusal surfaces, they must slide into place at an angle parallel to the axis of the tooth, or nearly so. In these cavities the floor of the cavity is generally below the gingival margin, which affords an excellent retention; but should it be level with the gingival margin it will be necessary, with a round bur, to cut a small groove in the floor to act as a retention at this point. The labial and lingual walls should be slightly grooved occlusal to form a mortise for retention. On the occlusal surfaces these grooves may be slightly flared to prevent distortion of the wax impression when withdrawn, and also causes the inlay when seated to fit tightly.

*Wax Impressions.*—Especially prepared wax in stick form can be obtained. After preparing the cavity, shape the end of the stick of wax to very near the size of the cavity, then slightly warm and press firmly into the wet cavity. Trim off the surface and have the patient bite into the wax; that is, if it is on the masticating surface. While the teeth are closed, with a small spatula, press and burnish the wax over the labial margins and when the mouth is open, with a slight pressure of the finger hold the wax into place, burnish the lingual margins and if possible, the gingival. Chill it with a few drops of cold water, and with a well vasolined celluloid strip contour and shape up the approximal surfaces as desired in the finished gold. Should some margins be a little short, small pieces may be added with a hot spatula. This may be withdrawn and the spru wire attached to the under surface, but you will find the best method is to attach the spru wire when the wax is in place. Should it be felt that there are any undercuts, and the filling not readily removed, by working the spru wire back and forth the wax is rubbed down, and the filling is felt to go in and out without any drag. Just before removing the wax for the last time, go over the margins to see if they are perfect. The back of the wax model should be thoroughly cleaned with a small camel's hair brush, as you will find small films of wax adhering to it, caused by the rubbing when the cavities happen to be slightly undercut.

*Investing.*—The best results will be obtained by investing the impression as soon after taking as possible, for if they are allowed to stand for a day or so in a warm room they are liable to distort and get out of shape. The best method is to take two or sometimes three impressions of the same cavity, as they are very easily made, and it does not require much time; so you will have an extra impression to fall back on, as you must expect to meet with some failures. The investing is best done with a small camel's hair brush, or a very fine spatula, putting a little of the investment on a smooth surface of the back of the impression, and then pushing it ahead to where you want it. This will exclude the air which causes trouble. Should bubbles form, do not jar out the bubble, but with your small camel's hair brush paint over it until it disappears. After thoroughly covering the wax with a thin coating allow it to stand so as to slightly harden before investing into the small flasks. The casting may be done by any of the different machine methods now on the market.

*Setting.*—After casting, cut off the stem and grind the inlay to the shape desired. If it is a simple case you can polish immediately, but if a difficult one, you will find it neceessary to fit it in the mouth first, and may have to be ground or touched up here and there to make it properly occlude, and then can be polished just before setting, as this does not take much time. Use the finest inlay cement of a golden brown color. In setting some inlays you will find it hard to exclude the air; this can be avoided by filling the cavity full of cement. With a burnisher, burnish the edges down while the cement is soft, and do not undertake to polish the edges until the cement is thoroughly set. Then test the bite with articulating paper, and grind to proper occlusion, if required.

